# Comparative epidemiology of influenza A and B viral infection in a subtropical region: a 7-year surveillance in Okinawa, Japan

**DOI:** 10.1186/s12879-016-1978-0

**Published:** 2016-11-08

**Authors:** Yoshikazu Iha, Takeshi Kinjo, Gretchen Parrott, Futoshi Higa, Hideaki Mori, Jiro Fujita

**Affiliations:** 1 Department of Infectious Diseases, Respiratory, and Digestive Medicine, Graduate School of Medicine, University of the Ryukyus, 207 Uehara, Nishihara, Okinawa 903-0215 Japan; 2 Department of Nursing, University of the Ryukyus Hospital, 207 Uehara, Nishihara, Okinawa 903-0215 Japan; 3 Department of Respiratory Medicine, National Hospital Organization Okinawa Hospital, 3-20-14 Ganeko, Ginowan, Okinawa 901-2214 Japan; 4 Clinical Laboratory Center, Medical Association of Naha City, 26-1 Higashimachi, Naha, Okinawa 900-0034 Japan

**Keywords:** Influenza B, Epidemics, Seasonality, School-age, Climate condition, Temperature, Humidity

## Abstract

**Background:**

The epidemic patterns of influenza B infection and their association with climate conditions are not well understood. Influenza surveillance in Okinawa is important for clarifying transmission patterns in both temperate and tropical regions. Using surveillance data, collected over 7 years in the subtropical region of Japan, this study aims to characterize the epidemic patterns of influenza B infection and its association with ambient temperature and relative humidity, in a parallel comparison with influenza A.

**Methods:**

From January 2007 until March 2014, two individual influenza surveillance datasets were collected from external sources. The first dataset, included weekly rapid antigen test (RAT) results from four representative general hospitals, located in the capital city of Okinawa. A nation-wide surveillance of influenza, diagnosed by RAT results and/or influenza-like illness symptoms, included the age distribution of affected patients and was used as the second dataset. To analyze the association between infection and local climate conditions, ambient temperature and relative humidity during the study period were retrieved from the Japanese Meteorological Agency website.

**Results:**

Although influenza A maintained high number of infections from December through March, epidemics of influenza B infection were observed annually from March through July. The only observed exception was 2010, when the pandemic strain of 2009 dominated. During influenza B outbreaks, influenza patients aged 5 to 9 years old and 10 to 14 years old more frequently visited sentinel sites. Although both ambient temperature and relative humidity are inversely associated with influenza A infection, influenza B infection was found to be directly associated with high relative humidity.

**Conclusion:**

Further studies are needed to elucidate the complex epidemiology of influenza B and its relationship with influenza A. In the subtropical setting of Okinawa, epidemics of influenza B infection occur from March to July following the influenza A epidemic, and primarily affect school-age children. These findings help to define unknown aspects of influenza B and can inform healthcare decisions for patients located outside temperate regions.

**Electronic supplementary material:**

The online version of this article (doi:10.1186/s12879-016-1978-0) contains supplementary material, which is available to authorized users.

## Background

Influenza A virus mutates frequently to escape the host immune system and often causes epidemics. Historically, this had led to pandemics. Thus, influenza A infection is regarded as important to both medical and social concerns [1, 2]. Alternatively, influenza B virus mutates less frequently than influenza A virus [3, 4]. Although some regional outbreaks are due to influenza B virus [5–8], the virus has never caused a documented pandemic. Consequently, most epidemiological studies have focused on influenza A infection rather than influenza B infection.

In temperate regions, it is well established that epidemics of influenza A often occur during the winter [3]. However, in tropical and subtropical regions, influenza A infections have been observed throughout the year [9]. In contrast, seasonal information for influenza B is limited and epidemic patterns of influenza B can vary among investigations [10, 11]. Again, most climate analysis studies have focused on influenza A infection; data regarding influenza B infection is lacking considerably [10, 11].

In Japan, the universal healthcare system supports all citizens to visit outpatient clinics, or the emergency departments of general hospitals, without referral. As a result, patients with mild disease, including upper respiratory tract infection, often visit general hospitals directly. In many cases, attending physicians suspecting an influenza-like illness (ILI) will use a rapid antigen test (RAT), which can detect influenza A and B separately [12]. In Okinawa, four representative general hospitals aggregate and report the weekly results of the RATs. Using this routine surveillance data, our investigative team previously reported the epidemic pattern of influenza A viral infection and its relationship to climatic conditions [13, 14]. This retrospective study aims to characterize the epidemic patterns of influenza B by combining the aforementioned RAT reported results with the nation-wide influenza surveillance data accumulated between 2007 and 2014. Our multicenter approach includes patients from Okinawa, an important surveillance region in subtropical Japan. Influenza A in this study is included for comparison.

## Methods

### Dataset 1- local surveillance of influenza virus infection by rapid antigen test (Additional file 1)

The clinical laboratories of four representative general hospitals, Naha City Hospital (470 beds), Okinawa Red Cross Hospital (314 beds), Okinawa Prefectural Nanbu Medical Center (434 beds), and Urasoe General Hospital (311 beds), in the capital city of Okinawa, reported the results of the RATs for influenza virus weekly to the Clinical Laboratory Center of the Medical Association. Data accessed from the Clinical Laboratory Center of the Medical Association included only the total number of tests administered and the number of positive influenza A, B, or A/B co-infected results. Patient information and symptomatology were not available.

### Dataset 2- Japanese national sentinel surveillance data (Additional file 1)

The Japanese national influenza surveillance is conducted in approximately 5000 sentinel healthcare facilities throughout Japan; 55 facilities are within the Okinawa Prefecture [15]. Data from these 55 sentinel healthcare facilities within Okinawa was extracted from the Infectious Diseases Weekly Reports, published by the National Institute of Infectious Diseases in Japan [16, 17]. This surveillance dataset includes the age distribution of patients diagnosed with an influenza infection based on either a positive RAT result and/or the presence of symptoms from ILI. Influenza-like illness is defined with four criteria as follows; 1) acute onset of symptoms, 2) high fever, 3) upper respiratory symptoms, and 4) general symptoms such as malaise, headache, and myalgia. Patients with either a positive RAT result or who meet all four ILI criteria can be added to the database.

### Evaluation of age distribution of influenza A or B infection

Weekly disease patterns were monitored in dataset 1 retrospectively. A week in which influenza A or B cases accounted for more than 90 % of all positive influenza cases was subsequently defined as an “epidemic week” for either influenza A or B, respectively. Weeks with no predominant influenza type (i.e., A to B ratio was 1:1) were not considered as “epidemic weeks” and were excluded from age distribution analysis. Weeks determined as “epidemic weeks” in dataset 1 were matched to the corresponding week in dataset 2. Patient age distribution from those corresponding weeks was extracted from dataset 2 for the sentinel sites located within the Okinawa Prefecture. A visual representation for this method is provided in Additional file 2: Table S1.

### Geographic and climatic background

Okinawa Island is located in the East China Sea approximately 640 km south of the rest of Japan, and roughly 500 km north of Taiwan. Possessing a diverse native population, a large population of semi-permanent foreigners, and an ample tourist trade, Okinawa has proven its worth as a surveillance site for the monitoring of circulating respiratory viruses prior to epidemic outbreaks in mainland Japan [18]. Average temperatures are 18 °C in winter and 28 °C in summer. The island’s subtropical climate supports a dense forest and a rainy season occurring in the late spring. Daily climate data including ambient temperature and relative humidity was retrieved from the Japanese Meteorological Agency website (http://www.jma.go.jp/jma/index.html) [19]. Weekly climate variables were calculated using the retrieved data.

### Statistical analysis

The duration of infectious activity was assessed using the maximum proportion of cases during consecutive weeks and the minimum number of weeks during which there were at least 80 % positive RAT results, a method previously described by Caini S, et al. [20]. Positive RAT cases and climate variables were evaluated by Spearman’s correlation coefficient test (one sided). The datasets used in this study can be found within the additional files. Statistical analyses were performed using the SPSS software (version 20.0, IBM Tokyo, Japan).

## Results

**Fig. 1:**
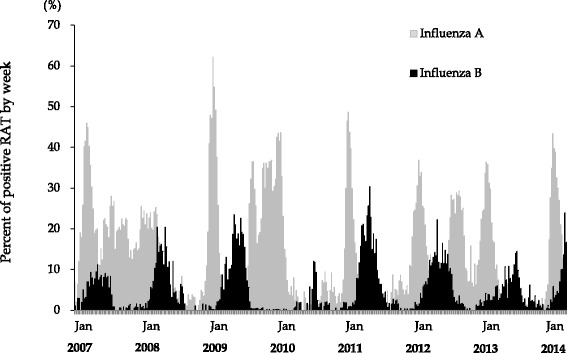
Epidemiology of influenza virus infection in Okinawa, Japan. Gray and black bars indicate the percent of positive RATs for influenza **A** and **B** infection among RATs performed, respectively, between 2007 and 2014 from four representative hospitals

**Fig. 2:**
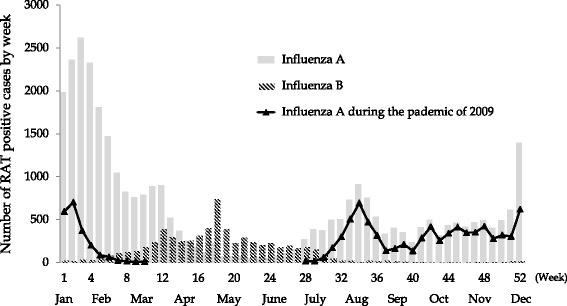
Pooled number of positive cases of influenza infection by week. Gray and hashed bars indicate the number of positive cases determined by rapid antigen test (RAT) for influenza **A** and **B** infection, respectively. Cases are pooled for each week from 2007 until 2014 using the RAT results from four representative hospitals. Triangles indicate the number of influenza **A** infections determined by RAT during the pandemic year of 2009 from four representative hospitals (from June 2009 to March 2010)

From January 2007 to March 2014, 168,874 RATs were performed within the four representative hospitals. The use of RATs diagnosed a total of 37,309 and 7277 influenza A and influenza B infections, respectively. The incidence of influenza B infection was lowest for 2010, the year following the influenza pandemic of 2009. The annual number of influenza B positive cases ranged from 107 (0.9 % of all RATs in 2010) to 1940 (9.5 % of all RATs in 2011), as shown in Table 1. Co-infection with both A and B was not common. Figure 1 shows the percentage of positive cases for influenza B was less than influenza A. Epidemic analysis of influenza B revealed bimodal peaks observed in March and May (data not shown). The middle 80 % of influenza B infections were seen from week 11 (middle March) to week 30 (end July), in contrast to the middle 80 % of non-pandemic strain influenza A cases occurring from week 51 (end December) to week 13 (middle March) of the following year. Influenza A infection, in seasons other than winter, was primarily attributed to the pandemic strain of 2009 (Fig. 2).

**Table 1:** Result of rapid antigen tests performed in four general hospitals in Okinawa

Year	Number of tests performed	Influenza A positive	Influenza B positive	Influenza A/B positive
2007	19,229	5143 (26.7 %)	623 (4.4 %)	6 (0.04 %)
2008	13,089	1878 (14.3 %)	797 (7.1 %)	4 (0.04 %)
2009	41,962	12,741 (30.4 %)	1153 (3.9 %)	18 (0.06 %)
2010	13,825	2508 (18.1 %)	107 (0.9 %)	2 (0.02 %)
2011	24,468	4128 (16.9 %)	1940 (9.5 %)	7 (0.04 %)
2012	27,148	5284 (19.5 %)	1353 (6.2 %)	4 (0.02 %)
2013	18,494	2475 (13.4 %)	728 (4.5 %)	0 (0.00 %)
2014 (Jan. to Mar.)	10,659	3152 (29.6 %)	576 (7.7 %)	1 (0.01 %)
Total	168,874	37,309 (22.1 %)	7277 (4.3 %)	42 (0.03 %)

The annual number of rapid antigen tests (RAT) performed and reported positive results for influenza A, B, and A/B co-infected patients in four representative hospitals in Okinawa, Japan

**Fig. 3:**
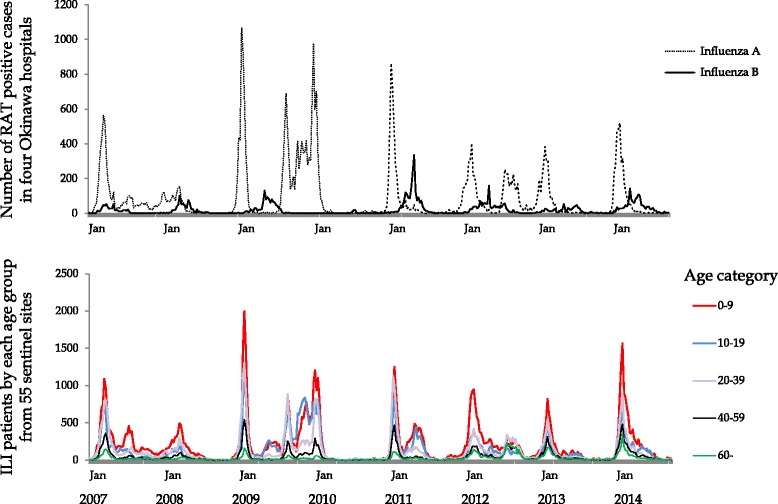
Matched analysis of the two datasets. Upper figure shows dataset 1 monitoring for the incidence of influenza **A** and **B** infection from four representative hospitals in Okinawa. Lower figure shows the distribution of each age group among influenza patients in dataset 2

**Fig. 4:**
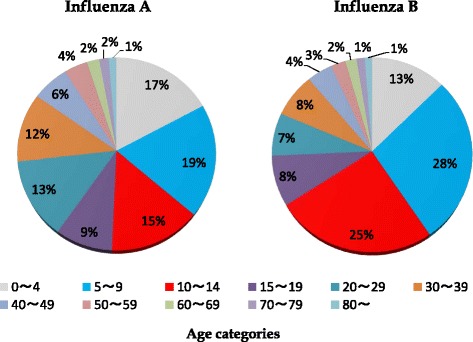
Age category distribution during epidemic weeks defined as influenza **A** or **B** infection. Epidemic weeks of influenza **A** or **B** were identified using the described method within dataset 1. Information regarding age distribution for all identified weeks was extracted from dataset 2. Assumed cases of influenza **A** and **B** totaled 148,244 and 13,606, respectively. Only patient data from the 55 sentinel sites located within Okinawa was used for age comparison

Epidemic curves from the two datasets were aligned and analyzed to define at risk age groups (Fig. 3). Overall, patients aged 0 to 9 years old or 10 to 19 years old more frequently visit the sentinel sites included in dataset 2 (lower) during periods of influenza B outbreaks reported in dataset 1 (upper). An additional analysis of age categories was conducted to validate these results. Age categories of the patients affected by either influenza type were determined using the method depicted in Additional file 2: Table S1. This method subsequently defined, 173 weeks with influenza A cases >90 % (148,244 assumed influenza A cases) and 78 weeks with influenza B cases >90 % (13,606 assumed influenza B cases). Age dynamics from weeks categorized as influenza B weeks show 5 to 9 year-olds (28 %) and 10 to 14 year-olds (25 %) as the predominant age groups seeking healthcare for confirmed or probable cases of influenza. The same age patterns were not observed for influenza A weeks (Fig. 4).

**Fig. 5:**
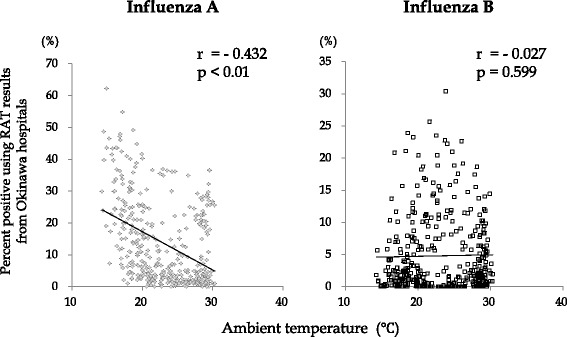
Ambient temperature’s effect on influenza infections in Okinawa, Japan. The relationship between ambient temperature and the percent of positive influenza **A** or **B** infection for RAT results performed in four representative hospitals was analyzed with Spearman rank correlation coefficient (one-sided). r; correlation coefficient, *p*; *p* value

**Fig. 6:**
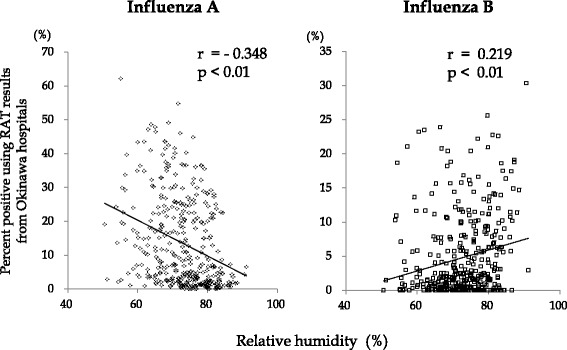
Relative humidity’s effect on influenza infections in Okinawa, Japan. The relationship between relative humidity and the percent of positive influenza **A** or **B** infection for RAT results performed in four representative hospitals was analyzed with Spearman rank correlation coefficient (one-sided). r; correlation coefficient, *p*; *p* value

Finally, the impact of climate variables on the proportion of positive influenza A or B results was evaluated on a weekly basis. As shown in Figs. 5 and 6, a higher proportion of influenza A positive cases were observed during periods of lower ambient temperature and lower relative humidity. Alternatively, a high proportion of influenza B positive cases were observed during times of high relative humidity. No association between ambient temperature and influenza B could be determined.

## Discussion

Okinawa’s annual influenza B epidemics primarily occur between March and July and display bimodal peaks in March and May. Although Kikuchi et al. reported a 2006 epidemic of influenza B from May to June in Sapporo, Japan [6], epidemics of influenza B infection are rarely reported from mainland Japan and other temperate regions. However, intermittent epidemics of influenza B have been reported from Taiwan [21]. Also, a recently published article by the Global Influenza B Study Team examined the cooperation and coordination of influenza A and B viruses. Together, influenza A and B’s combined effect successfully extended the influenza season for countries located within tropical and subtropical regions [20]. Similarly, the sub-tropical climate of Okinawa may enable the seasonal patterns of influenza infection observed in the present study.

It is common knowledge that cold, dry weather can produce favorable conditions for influenza A virus transmission [22–24]. It is also known that influenza A virulence is considered greater than that of influenza B virus due to genetic shift and drift [2, 25, 26]. Following a winter with influenza A infection in decline, it is possible, influenza B virus could more easily spread among those with weakened immunity. Additionally, the dynamics among viruses themselves can influence patterns of epidemics. As we have seen with the pandemic strain in 2009–2010, influenza A has the ability to suppress outbreaks of other respiratory viruses, particularly influenza B [27]. Thus, the differences in pathogenicity between influenza A and B could also explain the seasonal patterns observed in Okinawa. Ultimately, the characteristics of influenza epidemics for tropical and subtropical countries are complex and still poorly understood. In reality, the interaction of several climatic and ecological drivers, including temperature, humidity, altitude precipitation, population density and cultural mores or gathering events could create the ideal setting for pervasive circulation.

In the present study, we were able to assess the association between the incidence of influenza A or B infection and limited climatic conditions. Influenza A virus remained significantly associated with cold and dry climate conditions [22–24]. However, the association between influenza B infection and climatic conditions was less distinctive. Our analysis confirmed a high percentage of influenza A positive cases were observed during periods of lower ambient temperature and lower relative humidity. On the other hand, a high percentage of influenza B cases were associated with high humidity. No link was observed between influenza B infection and temperature (Figs. 5 and 6). In this study, we use relative humidity not specific humidity, which may limit the accuracy of our results. Previous investigations from our team have also used relative humidity [13, 14], and the standardization of the method was important to compare this current study with previous data. Recent articles have reported specific humidity is a better predictor of epidemics of influenza [28, 29]. Therefore, further analysis using specific humidity is needed to clarify the relationship between influenza B and humidity in Okinawa. Since multiple factors are involved in determining region-specific epidemic patterns of influenza infection [30], more prospective epidemiological studies, including analyses of meteorological factors, are needed to understand the circumstances found in Okinawa and other tropical and subtropical regions.

During influenza B epidemics, our results showed the primary patient group visiting healthcare facilities in Okinawa was school-age children (Figs. 3 and 4). Alternatively, influenza A affects a much broader age range, suggesting that this phenomenon is unique to influenza B infection. The pathogenicity of influenza A virus is considerable and all age-groups are susceptible to influenza A infection [3, 16]. As a result of this virulence, differences in prevalence among each age-group might become less distinct. Influenza B virus cannot cause massive epidemics among all age-groups due to its relatively weak virulence. Other reports also show that influenza B patients were younger than influenza A patients [10, 11].

Healthcare seeking behavior may also account for the different patterns observed among the age groups. It is well known that the school-age population can easily spread influenza infections [31–34]. Working adults might be less likely to visit a clinic with only mild symptoms due to influenza B infection. However, parents of school age children may be more likely to seek immediate care for a sick child, regardless of severity of symptoms. Additionally, acquired immunity within the host may account for the observed differences among the age groups. Adults with repeated exposure to influenza B may be less susceptible to the disease, since mutation rates for influenza B are not as rapid as influenza A [35].

This study has some limitations. First, sample collection and laboratory analysis were performed externally. The results of RATs performed in four representative general hospitals in Okinawa is routinely accumulated and summarized on a weekly basis. In addition, Japan has a nation-wide influenza surveillance system with weekly reports available to all physicians. All data was received either through the local prefectural influenza reporting system or through the Japanese national influenza reporting system. Due to this external processing, patient details such as gender, co-morbidities, and accompanying symptoms could neither be accessed nor analyzed. Nevertheless, the Western Pacific Region Global Surveillance and Response System has set precedent that surveillance data for ILI symptoms and datasets based on influenza viral detection can be synchronized [25]. Our data replicates this method and confirmed the epidemic curves of the two different datasets overlapped in Okinawa (Fig. 3).

A second limitation is the lack of standardization in RAT protocols and manufacturers. Due to retrospective nature of this study and the diversity of hospitals reporting data, there was no way to standardize which RAT test was implemented. Okinawa serves as an important surveillance point to monitor the circulating strains of influenza [13, 18]. Therefore, physicians in Okinawa are perpetually aware and use RATs for patients with ILI, regardless of the season. As a result, usage of RATs for patients with ILI is higher than average. Overall, the diagnostic accuracy of RATs sold in Japan is high. At one point in 2013, 17 different RATs for influenza virus were available in Okinawa. According to the included manufacturer’s documents, the sensitivity and specificity of RATs for influenza A virus, when compared with a gold standard viral culture, ranged from 87.9 to 100 % and 88.8 to 100 %, respectively. Detection of influenza B virus ranged from 80.4 to 100 % and 90.7 to 100 %, respectively. However, RAT results should be expected to show significant heterogeneity due to differences in patient age, virus type and brand variety, all of which can affect test results. Furthermore, some studies describe lower sensitivity for detection of influenza B infection than that of influenza A infection [12, 36–39]. This decrease in sensitivity may cause even lower rates of detection for influenza B, due to increased false negative results in some populations.

Lastly, this study did not investigate any patient factors with influence on immunity, mainly, vaccination. In Japan, a quadrivalent vaccine against influenza viruses was introduced in the 2015–16 season. However, the administration of influenza vaccine is generally recommended before winter, and it is doubtful if the efficacy of vaccine against influenza B infection lasts until spring. In the future, discussions regarding the efficacy and the cost effectiveness of the single quadrivalent vaccine compared to a bi-annual vaccine schedule should be considered if a decrease in influenza infections is not achieved in Okinawa.

## Conclusion

In Okinawa, influenza B epidemics primarily occur from March until July with bimodal peaks in March and May and predominately affect school-age children. Our data suggests high relative humidity may increase rates of influenza B infection. No novel information was discovered regarding our comparator, influenza A, from this study. Additional studies within the surveillance site of Okinawa, or other subtropical regions are needed to confirm our results and expand our knowledge regarding the epidemiology of influenza B.

## Additional files

Additional file 1: Dataset 1. Positivity rate. Dataset 2. Patiant’s age (XLSX 161 kb)
Additional file 2: Table 1. Example weeks from dataset 1 and dataset 2 post-alignment. Sample weeks shown here are to serve as a visual representation of the method outlined to evaluate age distribution. Dataset 1 (left) and dataset 2 (right) were combined and aligned following the selection of “epidemic weeks”. A week in which influenza A or B cases accounted for more than 90 % of all positive influenza cases was defined as an epidemic week. In the year 2007, you see an example of a defined influenza A epidemic week, whereas the year 2011 is a representative influenza B epidemic week. The week from 2013 displays a typical week which was removed from our age distribution analysis because neither influenza A nor B was dominant (>90 %). (PPTX 66 kb)

## Abbreviations

ILI: Influenza-like illness
RAT: Rapid antigen test
