# Supplementary material for: Comparative epidemiology of influenza A and B viral infection in a subtropical region: a 7-year surveillance in Okinawa, Japan
Source: BMC Infect Dis. 2016 Nov 8;16:650. doi: 10.1186/s12879-016-1978-0 (PMC5100171; doi:10.1186/s12879-016-1978-0)
Supplement: Additional file 2: Table 1. — Example weeks from dataset 1 and dataset 2 post-alignment. Sample weeks shown here are to serve as a visual representation of the method outlined to evaluate age distribution. Dataset 1 (left) and dataset 2 (right) were combined and aligned following the selection of “epidemic weeks”. A week in which influenza A or B cases accounted for more than 90 % of all positive influenza cases was defined as an epidemic week. In the year 2007, you see an example of a defined influenza A epidemic week, whereas the year 2011 is a representative influenza B epidemic week. The week from 2013 displays a typical week which was removed from our age distribution analysis because neither influenza A nor B was dominant (>90 %). (PPTX 66 kb) [file 12879_2016_1978_MOESM2_ESM.pptx]

## Slide 1
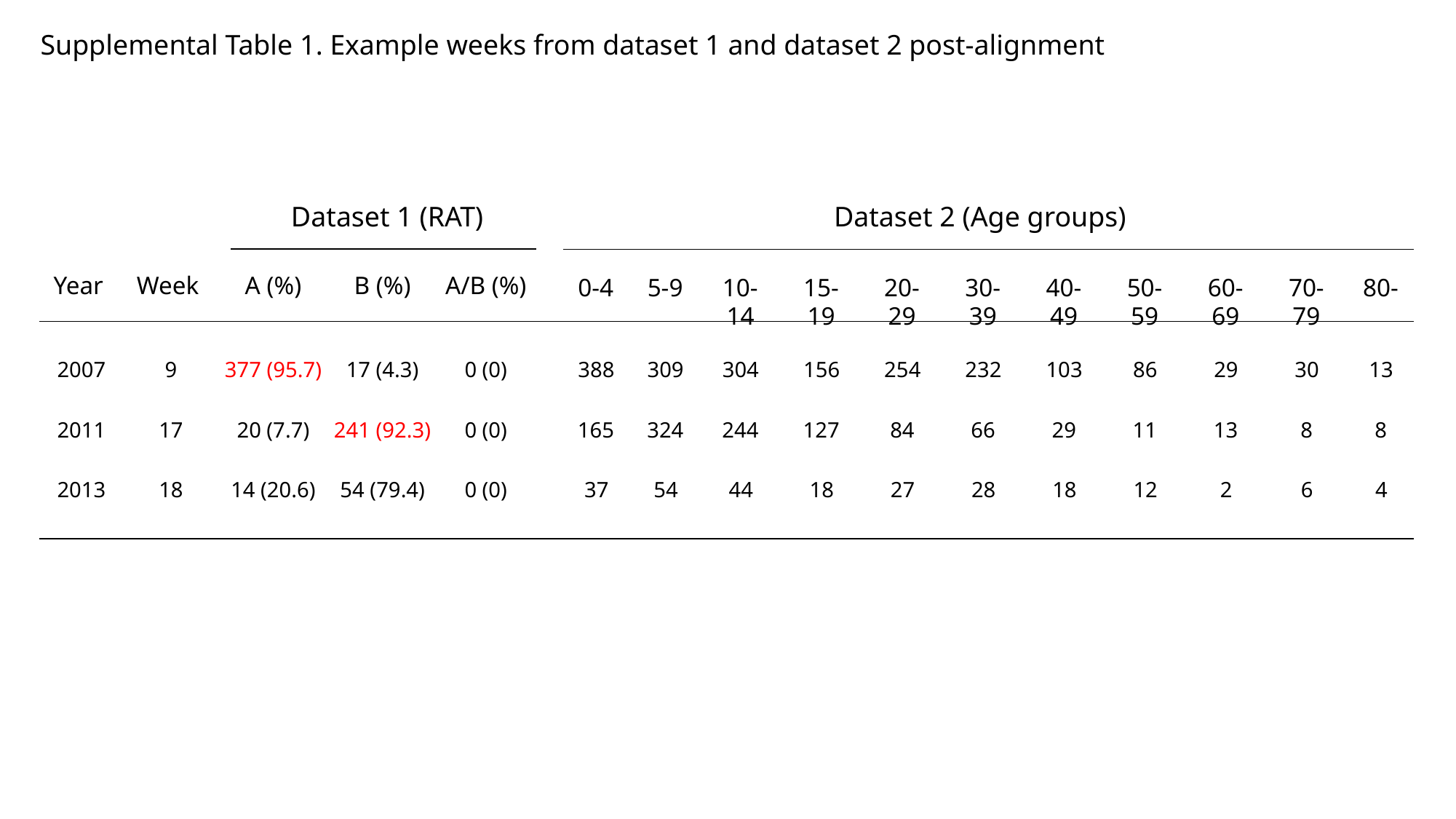

Supplemental Table 1. Example weeks from dataset 1 and dataset 2 post-alignment
Dataset 1 (RAT)
Dataset 2 (Age groups)
Week
A (%)
B (%)
A/B (%)
Year
0-4
5-9
10-14
15-19
20-29
30-39
40-49
50-59
60-69
70-79
80-
2007
9
377 (95.7)
17 (4.3)
0 (0)
388
309
304
156
254
232
103
86
29
30
13
2011
17
20 (7.7)
241 (92.3)
0 (0)
165
324
244
127
84
66
29
11
13
8
8
2013
18
14 (20.6)
54 (79.4)
0 (0)
37
54
44
18
27
28
18
12
2
6
4
